# A feasibility study on the application of MICRO XRF for latent fingermark detection on porous surfaces

**DOI:** 10.1111/1556-4029.70221

**Published:** 2025-11-14

**Authors:** Sang‐Yoon Lee, Sae‐Hee Yang, Seung‐Hee Kang, Ki‐Jong Rhee

**Affiliations:** ^1^ Department of Forensic Sciences Yonsei University Wonju Republic of Korea; ^2^ Department of Biomedical Laboratory Science Yonsei University Wonju Republic of Korea

**Keywords:** latent fingermark, MICRO XRF (X‐ray fluorescence), porous substrate

## Abstract

In the context of criminal investigations, latent fingermarks play a pivotal role in obtaining clues related to suspects. Presently, various physical, chemical, and optical methods are employed for latent fingermark detection. However, it is observed that when utilizing physical and chemical techniques, latent fingermarks may sometimes suffer damage during the extraction process. Consequently, the importance of optical methods such as spectroscopy, ATR‐FTIR, and MICRO XRF, which are comparatively less destructive, has been on the rise these days. This study aimed to evaluate the applicability of MICRO XRF for detecting both natural and artificial latent fingermarks on porous paper surfaces. Natural latent fingermarks were deposited by five donors after handwashing, while artificial latent fingermarks were produced by printing an artificial fingermark solution. MICRO XRF successfully captured elemental signals, with chlorine and potassium providing the clearest images for natural fingermarks and chlorine for artificial fingermarks. These findings demonstrate the potential of MICRO XRF to image latent fingermarks nondestructively regardless of background color. This work lays the foundation for further research to refine artificial formulations, optimize acquisition parameters, making it a promising choice for prioritizing latent fingermark detection methods.

**Highlights**

- MICRO XRF was successfully applied for latent fingermark detection on porous surfaces such as paper.
- This method provides chemical‐specific, nondestructive imaging of latent fingermarks without traditional treatments.
- An artificial latent fingermark solution was tested to simulate realistic deposition conditions.
- MICRO XRF serves as a complementary tool in forensic workflows for fragile or complex substrates.

## 1 INTRODUCTION

Fingermark evidence at a crime scene is a crucial piece of evidence for identifying suspects. People come into contact with various surfaces as they go about their daily lives, and their fingermarks can become contaminated with a variety of substances [1, 2, 3]. When a fingermark is deposited on a surface, it commonly contains amino acids and lipids, with variations in their composition depending on an individual's diet and personal characteristics. Additionally, fingermarks can incorporate elements from the contacted surface, apart from amino acids and lipids [2, 3, 4]. The components of deposited fingermarks are vital for visualization. Fingermark residues consist of substances produced by the epidermis and dermis of the skin when excluding contaminants from the outside world, mainly comprising amino acids and lipids, which are secreted by sweat and sebaceous glands [5]. Among these, sodium chloride, one of the constituents included in sweat, is the most abundant component [6]. The detected constituents in a deposited fingermark can be categorized into endogenous and exogenous substances. Among the various constituents detected, sodium (Na), chlorine (Cl), potassium (K), and calcium (Ca) are found commonly in fingermarks, representing the endogenous substances originating from sweat pores and eccrine sweat. Zinc (Zn), which is also endogenous, is produced in minute quantities in the human body and is challenging to differentiate from exogenous contaminants [2, 3, 4, 5, 7, 8].

Common methods for fingermark detection include physical, chemical, and optical techniques [9]. Physical methods often involve the use of fluorescent or magnetic powders, sometimes in combination with optical methods. Chemical methods utilize reagents such as Ninhydrin and 1,2‐Indandione (1,2‐IND), sometimes in conjunction with optical techniques. These two approaches are the most widely used and researched. However, these methods can yield variable results in fingermark detection due to the presence of fingermark residues, making them less effective in complex backgrounds or situations where fingermarks may be destroyed. As a result, recent advances have introduced nondestructive optical techniques like spectroscopy, attenuated total reflectance‐Fourier transform infrared (ATR‐FTIR), and micro X‐ray fluorescence (XRF) [4, 10]. Spectroscopy, such as ATR‐FTIR, can detect lipid response bands for fingermark analysis [11, 12]. However, obtaining clear and high‐quality fingermark images can be challenging, leading to the exploration of various X‐ray techniques for fingermark development [1, 2, 3]. To obtain high‐quality images, the quality of X‐ray beams is crucial. In the case of MICRO XRF, X‐rays are generated within an X‐ray tube and focused by optics for investigation. These X‐rays interact with the material being examined, causing fluorescence in the process. The emitted fluorescence is collected by an X‐ray fluorescence detector, and the collected signals are visualized as images based on sensitivity [7, 13].

In South Korea, there have been no studies or cases of fingermark development using MICRO XRF, and while previous studies have demonstrated the utility of MICRO XRF in visualizing latent fingermarks—mainly on non‐porous substrates—its application to porous materials has remained limited. Therefore, this study addresses that gap by exploring the feasibility of MICRO XRF for fingermark detection on porous substrates, and we attempted to develop fingermarks using MICRO XRF as a relatively nondestructive approach before any chemical or physical methods. Additionally, we attempted to detect fingermarks on surfaces commonly encountered in daily life.

## 2 MATERIALS AND METHODS

### 2.1 MICRO XRF

In this study, two MICRO XRFs were applied (M4 TORNADO+, BRUKER, Germany, and ATTOMAP, SIGRAY, USA). These systems were selected based on their availability and active use in South Korean forensic laboratories, where they currently represent the only MICRO XRF instruments accessible for such applications. In addition, both platforms are widely cited in international research involving elemental imaging and latent fingermark visualization, making them suitable benchmarks for comparative analysis. The M4 TORNADO+ and SIGRAY ATTOMAP systems used in this study were equipped with Ag and Rh anode tubes, respectively. For imaging, an Ag tube and an Rh tube were used. Chlorine elemental images were acquired using the Ag tube, and Potassium elemental images were acquired using the Rh tube. The Ag tube (Kα ≈ 22.16 keV) and the Rh tube (Kα ≈ 20.22 keV) were used according to the manufacturer's specifications. These spectral differences were considered in selecting imaging parameters to optimize elemental contrast for fingermark visualization. The M4 TORNADO+ with an Ag tube and a polycapillary lens, experiments were conducted at 50 kV and 300 μA. Both pure latent fingermarks and artificial latent fingermarks were imaged under the following conditions: Pixel size: 25 μm, Pixel time: 25 ms/pixel, and a measurement time of 33 hours and 8 minutes. For the ATTOMAP device, two types of anodes, Rh (L‐line) and chromium, were used, along with a 4 keV 2:1 Ellipsoidal Mirror. Experiments were conducted at 25 kV and 1200 μA. Pure latent fingermarks were imaged with a step size of 50 μm, a dwell time of 0.200 s, and a total measurement time of 20 h and 3 min. Artificial latent fingermarks were imaged under the same step size of 50 μm, a dwell time of 0.500 s, and a total measurement time of 22 h and 14 min. The X‐ray beam spot size used was <20 μm according to the manufacturer's specifications.

### 2.2 Fingermark preparation

For porous surfaces, uncontaminated A4 paper was prepared and cut into 4 × 4 cm pieces for use. To print artificial latent fingermarks, an artificial latent fingermark solution was created [14]. The prepared artificial latent fingermark solution was injected into an empty cartridge and attached to the printer. The artificial fingermarks were then uniformly printed using a printer (PIXMA iX6870, Canon, Japan). To create pure latent fingermark samples, 5 donors (2 females, 3 males in their 20s–30s) washed their hands with soap and allowed them to dry naturally until they were completely dry, which took approximately 15 min. The donors were instructed not to touch any other surface during this period. Afterward, they pressed their thumb onto the surface with an applied pressure of 10 N for 2 seconds, creating a total of 5 pure latent fingermarks.

## 3 RESULTS

**FIGURE 1:**
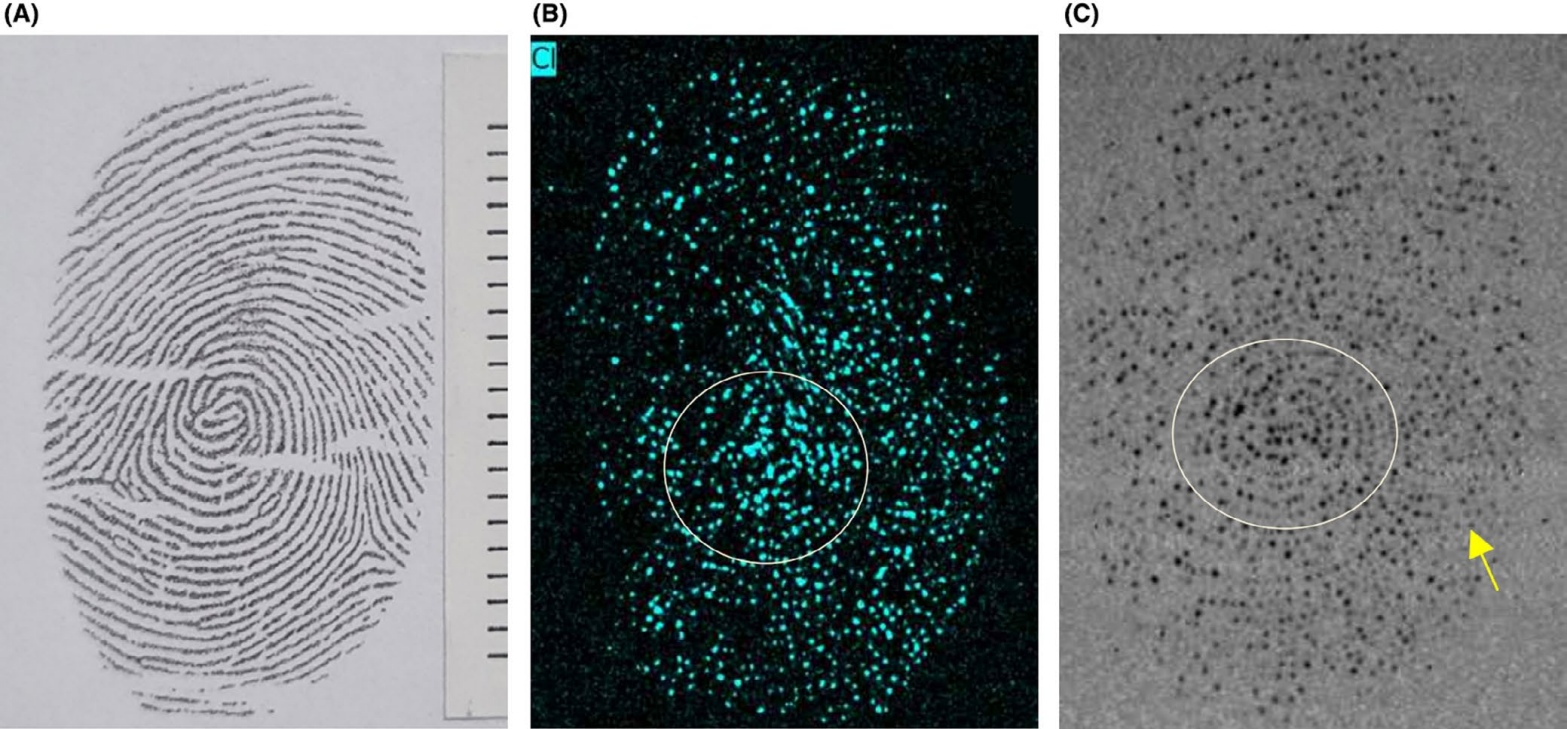
Pure latent fingermarks on paper imaged with MICRO XRF. (a) Reference fingermark, (b) Pure latent fingermark imaged with chlorine element (Ag tube), (c) Pure latent fingermark imaged with potassium element (Rh tube).

**FIGURE 2:**
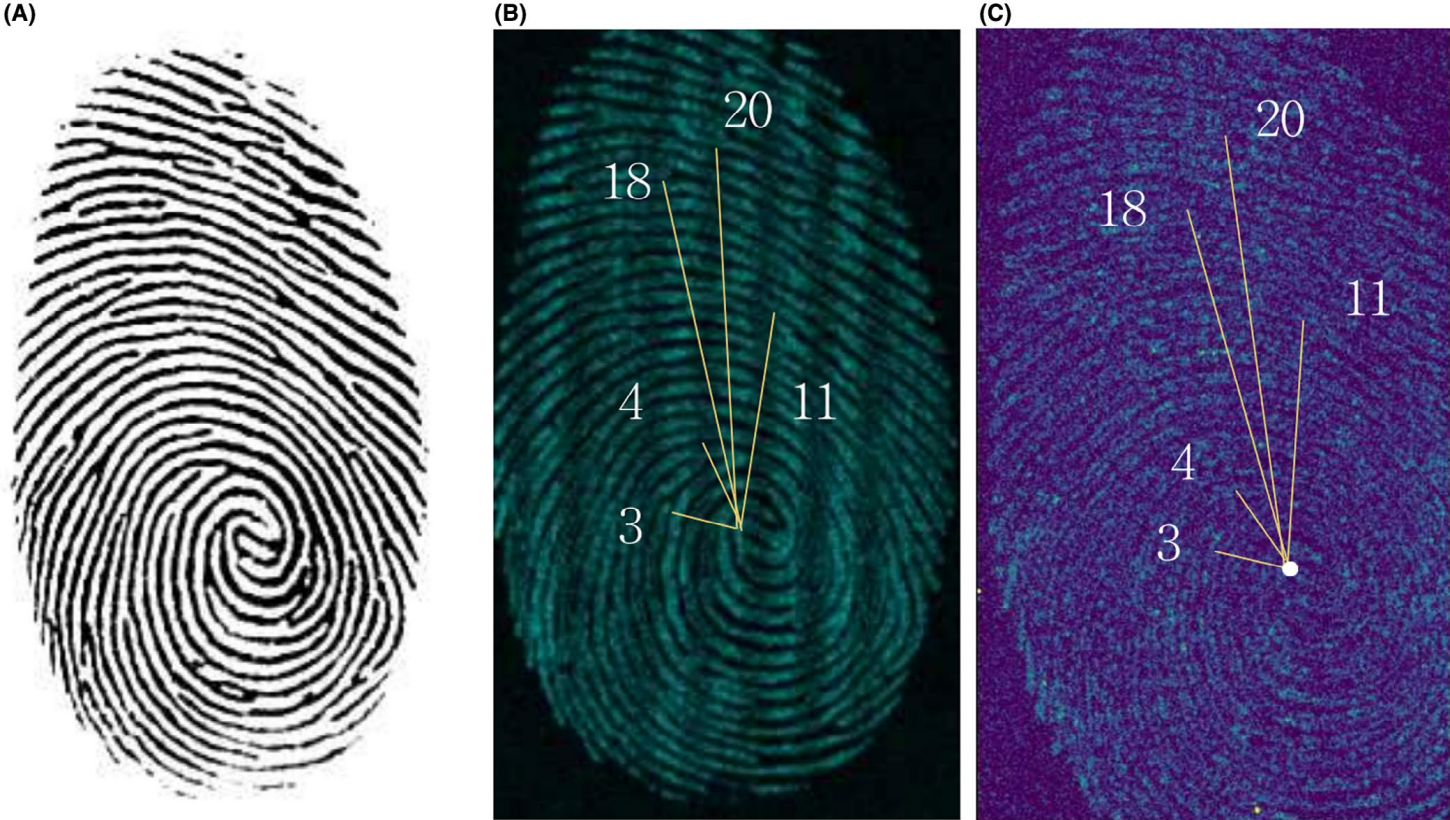
Artificial latent fingermarks printed on paper imaged with MICRO XRF. (a) Reference fingermark, (b) Artificial latent fingermark imaged with chlorine element (Ag tube), (c) Artificial latent fingermark imaged with potassium element (Rh tube).

In the case of pure latent fingermarks imaged with the silver tube, the images of sodium, calcium, and potassium did not provide clear differentiation of the fingermark ridges, but chlorine was successfully imaged (Figure 1B). For pure latent fingermarks imaged with the rhodium tube, sodium, chlorine, and calcium images did not show clear ridge differentiation, but potassium imaging allowed for the observation of the fingermark (Figure 1C). In the artificial latent fingermarks printed on paper, chlorine imaging was the most distinct among the analyzed elements. Potassium was included in the imaging analysis for comparison with natural fingermarks; however, since it was not present in the artificial formulation, no potassium signal was observed. All images represent individual elemental maps. Artificial latent fingermarks exhibited significantly stronger signals for sodium (Na) and chlorine (Cl), attributable to the high sodium chloride concentration (113 mM) in the artificial formulation (Table 1). This resulted in clearer and more uniform ridge visualization compared to natural fingermarks, which showed lower and more variable elemental signals due to individual physiological differences. Fingermark images were evaluated and categorized using a self‐established evaluation criteria table (Table 2). In the case of pure latent fingermarks, the core of the fingermark was observable, and in the images imaged using chlorine, the core of the pure latent fingermark was more distinct compared to those imaged using potassium. Additionally, chlorine‐imaged pure latent fingermarks exhibited blurry ridge patterns without observable minutiae, while potassium‐imaged pure latent fingermarks revealed some ridge details and vague minutiae such as bifurcations and endpoints. These differences are described further in the Discussion. For artificial latent fingermarks, the number of ridges from the core matched that of the reference fingermark, and it was possible to observe ridge bifurcations and endpoints (Figure 2). It should be noted that, unlike natural fingermarks, where eccrine sweat naturally diffuses from the pores along the ridge lines leading to variable elemental distributions, the artificial fingermarks were printed with a controlled solution, ensuring a consistent concentration of each element along the ridge lines, which may account for the clearer observation of ridge bifurcations and endpoints.

**TABLE 1:** Composition of artificial latent fingermark solution [14].

Component	Quantity	Concentration
*Amino acid solution*		
Serine	490 mg	9.3 mM
Glycine	294 mg	7.8 mM
Alanine	147 mg	3.3 mM
Lysine	195 mg	2.7 mM
Threonine	73 mg	1.2 mM
Asparagine acid	73 mg	1.1 mM
Histidine	73 mg	0.9 mM
Valine	49 mg	0.8 mM
Leucine	49 mg	0.7 mM
NaCl (sodium chloride)	3300 mg	113 mM
Magnesium chloride	4 mg	0.08 mM
Calcium chloride	16 mg	0.29 mM
Zinc chloride	2 mg	0.03 mM
Distilled water	500 mL	—
*Lipid solution*		
Palmitic acid	1.87 g	0.74 M
Oleic acid	1.0 mL	0.32 M
Stearic acid	0.80 g	0.28 M
Squalene	5.8 uL	1.21 mM
Cholesterol	7.46 mg	1.93 mM
Distilled water	9 mL	—

**TABLE 2:** Evaluation and classification of latent fingermarks.

Score	Categories
1	Impossible to confirm characteristic points and ridges, or latent fingermarks are not revealed
2	Difficult to distinguish between characteristic points and ridges
3	Characteristic points and ridges are blurred
4	Characteristic points are distinguishable, but some ridges are blurred
5	Characteristic points and ridges are clearly distinguishable

## 4 DISCUSSION

The results of latent fingermark development obtained using MICRO XRF are in the form of image files, allowing adjustments of parameters such as brightness and contrast to enhance the visibility of fingermarks without causing damage [1]. Furthermore, it is anticipated that fingermarks found at actual crime scenes often contain not only pure latent fingermark components but also residues from other substances or objects with which the fingermarks have come into contact. These transferred exogenous materials may result in the imaging of fingermarks with various elements, and it is believed that the strong signals from these transferred exogenous materials can contribute to obtaining high‐quality images [2]. The use of two MICRO XRF systems enabled a side‐by‐side comparison under realistic forensic conditions. While both provided consistent ridge detection, subtle differences in contrast and clarity—likely due to differences in X‐ray source and acquisition parameters—were observed. These findings highlight the need to consider system‐specific factors when applying MICRO XRF to latent fingermark detection. Currently, widely used physical and chemical methods in fingermark development heavily rely on background color. However, MICRO XRF has an advantage in producing clear fingermark images when the surface and the components contained in the fingermark exhibit sharp contrasts [2]. This is particularly advantageous when the components on the surface and within the fingermark differ significantly in terms of their elemental composition. In this study, although correction algorithms for background scattering or elemental noise were not applied, clean, uncontaminated paper was used, and imaging parameters were optimized to reduce scatter and enhance contrast. Although no correction algorithms were applied in this study, future work will explore background correction methods to improve image reliability on porous materials. While this study qualitatively discusses the differences in material distribution, a quantitative comparison of concentrations between natural and artificial latent fingermarks lies beyond its scope and warrants further investigation.

A limitation of this study is the relatively small number of five donors, which restricts the generalizability of the findings. Although donor‐specific comparisons were made, no significant differences were observed, and therefore, a representative dataset was presented. Future work with a larger and more diverse donor pool will be required to confirm the reproducibility of the observed imaging patterns. As noted in the Results, chlorine‐imaged pure latent fingermarks exhibited blurry ridge patterns with no observable minutiae, whereas potassium‐imaged fingermarks allowed for the identification of some ridge details and vague minutiae. This difference is likely related to the relatively short 15‐minute replenishment period after handwashing, which may have been insufficient for uniform residue redistribution across ridge lines, thereby affecting the visibility of certain ions. It is also important to acknowledge that the artificial fingermarks in this study were printed from a homogeneous solution, resulting in a consistent concentration of each element along the ridge lines. In contrast, natural fingermarks depend on eccrine sweat secretion and lateral diffusion from pores, leading to a more uneven distribution of residues across the ridges. This fundamental difference in deposition likely contributed to the clearer and more uniform appearance of artificial fingermarks compared to the natural samples. It is expected that adjusting the amount of the solution relative to the fingermark area can influence the quality of fingermark development. Future studies will also explore variations in sodium chloride concentration to assess the detection limits of MICRO XRF.

Although MICRO XRF has not yet been applied to latent fingermarks in South Korea, its use has been actively explored in international forensic research [1, 2]. X‐ray‐based techniques are valued for their nondestructive nature and ability to provide high‐resolution elemental or structural data. MICRO XRF has visualized latent fingermarks on various substrates by detecting residues such as sodium, potassium, and chlorine. This study builds on these international efforts to explore its applicability in the Korean forensic context. This research direction addresses practical issues in the field of forensic science, especially in the context of criminal investigations.

Previous studies have suggested that longer acquisition times can improve ridge clarity in captured fingermark images [9, 13]. However, prolonged acquisition may delay suspect identification, limiting practical on‐site use. Further evaluation is needed to determine optimal acquisition times for each substrate type, balancing image quality with operational feasibility.

In addition, optimizing fingermark imaging with MICRO XRF, adjusting images using brightness and contrast, considering the influence of exogenous substances, and evaluating the impact of solution quantity in artificial fingermark printing are all crucial steps toward improving the quality and accuracy of fingermark analysis [6, 14, 15].

## 5 CONCLUSION

In this experiment, the MICRO XRF application was successful in confirming the imaging of pure latent fingermarks and artificial latent fingermark ridges on porous surfaces such as paper. Furthermore, the X‐ray tube used in this experiment was found to be suitable for obtaining ridge images from deposited pure latent fingermarks and artificial latent fingermarks printed on paper. The results of the experiment showed that more ridge features could be identified in artificial latent fingermarks compared to pure latent fingermarks, and the ridge patterns in the images of artificial latent fingermarks were clearer (Table 3).

**TABLE 3:** Median (range) fingermark evaluation scores based on MICRO XRF (*n* = 5 fingermarks per condition).

Categories			Score	Categories			Score
BRUKER TORNADO (Ag tube)	Paper	Pure Latent fingermarks	3 (2–4)	SIGRAY ATTOMAP (Rh tube)	Paper	Pure latent fingermarks	4 (3–5)
		Artificial latent fingermarks	5 (4–5)			Artificial latent fingermarks	5 (4 = 5)

It is important to note that when applying MICRO XRF, the consideration of X‐ray fluorescence quantum yield plays a crucial role in imaging. Even with a high atomic number, if there i s not a sufficient amount of residue in the fingermark, acquiring a signal can be challenging. X‐rays are particularly responsive to metal ions, so if the surface is composed of metal ions, it may not yield a strong signal for fingermark imaging. Additionally, when dealing with curved surfaces, signal distortion reaching the detector can pose challenges for fingermark development. The choice of X‐ray conditions, such as size, thickness, and density of the sample, can vary depending on the composition of the surface, thickness, and density of the fingermark residue. In this study, the X‐ray conditions were adjusted to suit the characteristics of each fingermark sample, considering differences in residue composition, the degree of residue deposition, and the residue concentration.

## CONFLICT OF INTEREST STATEMENT

The authors declare no conflicts of interest.

## Data Availability

The data that support the findings of this study are available from the corresponding author upon reasonable request.
